# EGFR-targeting drugs in combination with cytotoxic agents: from bench to bedside, a contrasted reality

**DOI:** 10.1038/sj.bjc.6604373

**Published:** 2008-05-27

**Authors:** G Milano, J-P Spano, B Leyland-Jones

**Affiliations:** 1 Oncopharmacology Unit, Centre Antoine-Lacassagne, 33 Avenue de Valombrose, Nice 06189, France; 2 Medical Oncology Department, Hôpital Pitié Salpétrière, AP-HP, 47-83 Boulevard de l'Hôpital, Paris 75651, France; 3 Winship Cancer Institute, Emory University School of Medicine, 1365-C Clifton Road, Suite 40141, Atlanta, GA 30322, USA

**Keywords:** EGFR-targeting drugs, cytotoxic agents, combination, bench to bedside

## Abstract

The clinical experience recently reported with epidermal growth factor receptor (EGFR)-targeting drugs confirms the synergistic interactions observed between these compounds and conventional cytotoxic agents, which were previously established at the preclinical stage. There are, however, examples of major gaps between the bench and the bedside. Particularly demonstrative is the failure of the tyrosine kinase inhibitors (TKIs) (gefitinib and erlotinib) combined with chemotherapy in pretreated nonsmall cell lung cancer patients. These discrepancies can be due to several factors such as the methodology used to evaluate TKI plus cytotoxic agent combinations in preclinical models and the insufficient consideration given to the importance of the drug sequences for the tested combinations. Recent advances in understanding the biologic basis of acquired resistance to these agents have great potential to improve their clinical effectiveness. The purpose of this review is to critically examine the experimental conditions of the preclinical background for anti-EGFR drug–cytotoxic agent combinations and to attempt to explain the gap between clinical observations and preclinical data.

The epidermal growth factor receptor (EGFR) is present in many cell types and can be considered as one of the best-characterised ligand-receptor systems (Mendelsohn and Baselga, 2006). Epidermal growth factor receptor is a 170-kDa cell surface glycoprotein containing three well-identified domains: an extracellular ligand-binding domain, a hydrophobic membrane-spanning domain and a cytoplasmic portion containing the tyrosine kinase activity. The hyperactivation of EGFR signalling in tumours occurs via independent or combined mechanisms: overexpression of the receptor itself, autocrine overproduction of ligand with mainly EGF and tumour growth factor (TGFα), and EGFR mutations, notably through the variant III that maintains the EGFR signalling pathway in a state of continuous activation. Binding of a growth factor to its receptor initiates organised and oriented biochemical intracellular events. These include the activation of the receptor, a cascade of phosphorylation with different protein kinases and, at the nuclear level, the activation of transcription factors. The effects of EGFR activation on tumour cells are multiple and convergent, thus favouring uncontrolled cell growth with an increase in cell mobility and cell proliferation, a decrease in apoptotic machinery and stimulation of angiogenesis. In the clinic, EGFR overexpression has been associated with chemoresistance, disease progression and poor survival (Baselga, 2002). Considering the set of therapeutic tools targeting EGFR (Castillo *et al*, 2004), there are two well-identified categories of drugs, with monoclonal antibodies (mAbs) on one hand and tyrosine kinase inhibitors (TKIs) on the other. Both treatment tools have reached a mature stage of clinical development. Current therapeutic applications with anti-EGFR drugs show encouraging clinical results. This is true mainly for combinations with conventional cytotoxic agents. The rationale of these combinations derives from preclinical studies where additive and supra-additive interactions have been reported. In agreement with Harari *et al* (2007), it can be considered that EGFR signalling inhibition combined with radiation and chemotherapy have opened promising perspectives. But a significant part of patients in clinical trials do not demonstrate a favourable response. The purpose of this review is to critically examine the experimental conditions of the preclinical background for anti-EGFR drug—cytotoxic combinations and to attempt to explain the gap between clinical observations and preclinical data.

## Pharmacological consequences of EGFR targeting

The outcome of EGFR targeting is characterised by the disruption of a number of cellular processes that mirror the physiological consequences of EGFR signal transduction at the level of cell division, apoptosis and angiogenesis (Castillo *et al*, 2004). The monoclonal antibody C225 (cetuximab) has been shown to slow down cell division with changes in molecular actors controlling cell-cycle checkpoints (Kiyota *et al*, 2002). Similar observations were reported with the TKI ZD1839 (gefitinib) (Huang *et al*, 2002), in addition to its ability to modulate mechanisms of drug resistance, specifically the expression of ATP-binding cassette (ABC) transport proteins mediated via the MDR1 gene (Smith *et al*, 2008). It has been shown that both mAbs and TKIs are able to modify the cellular levels in apoptosis-related proteins underlying the pro-apoptotic effect of EGFR targeting (Ciardiello *et al*, 2000a; Baselga, 2001). Similarly, a recent study has demonstrated that the erlotinib-induced cell growth inhibition was accompanied by G_1_/S phase arrest, predominantly by suppression of G_1_/S-related cyclins and upregulation of the CDK inhibitor p27^KIP1^ (Ling *et al*, 2007). A negative influence of EGFR targeting on angiogenic biochemical mediators has been shown for both mAbs and TKIs; for instance, the tumour labelling in VEGF (vascular endothelial growth factor) and factor VIII was reduced in xenograft models by C225 (Huang and Harari, 2000) as well as by ZD1839 (Tortora *et al*, 2001). Interestingly, and perhaps insufficiently emphasised, is the fact that EGFR signal abrogation may lead to a diminution of key mechanisms in DNA repair (Bandyopadhyay *et al*, 1998). In this latter study, confocal imaging demonstrated that a significant proportion of DNA protein kinase was colocalised with EGFR in C225-EGFR targeted cells.

Logically, this above-mentioned pharmacological background justified combining EGFR-targeting drugs with conventional cytotoxic agents, in anticipation of supra-additive antitumour effects. It must be underlined at this level that there is a marked difference in the magnitude of antitumour effects in favour of cytotoxic drugs as compared with EGFR-targeting agents.

## EGFR targeting plus conventional cytotoxic agents

### From the bench

On the basis of current knowledge, there is no apparent distinction between TKIs and mAbs regarding their propensity to trigger, in the majority of cases, synergistic cytotoxic interactions with chemotherapeutic agents or irradiation. To a great extent this synergy can be attributed to the well-identified impact of EGFR-targeting drugs on cell division, apoptosis, angiogenesis and DNA repair (see above). In addition, the occurrence of cross-resistance is infrequent as the cellular targets and mechanisms of action of cytotoxic drugs and EGFR-targeting therapeutics are different. It must be stressed, however, that there are few, if any, experimental studies designed to thoroughly explore the different anticancer agents, class by class, in association with EGFR-targeting drugs and using appropriate methods of analysis (the Chou and Talalay model, for instance). An exception is the study by Ciardiello *et al* (2000b), who undertook to combine ZD1839 (gefitinib) and a panel of anticancer agents including platinum derivatives, taxanes, doxorubicin, etoposide, topotecan and raltitrexed. Treatments combining cytotoxic drugs and ZD1839 produced tumour growth arrests in established GEO human colon cancer xenografts, whereas, in single-agent-treated mice, tumours resumed growth similar to controls. On comparable experimental bases, Sirotnak *et al* (2000) reached similar conclusions when combining ZD1839 and taxanes, whereas associations with gemcitabine or vinorelbine led to more contrasting results. When combining gemcitabine and PKI 166, Kedar *et al* (2002) found convincing evidence of supra-additivity in human renal cell carcinoma growing orthotopically in nude mice. We reported on the association between ZD1839 and cisplatin-5-fluorouracil (5-FU) in head and neck cancer cell lines, which demonstrated the presence of sequence-dependent synergistic cytotoxic effects (Magné *et al*, 2002). Synergistic interaction between cisplatin and TKIs was also observed with CI-1033, an irreversible pan ErbB TKI (Gieseg *et al*, 2001). Erlotinib, combined with chemotherapy, has also been studied in preclinical models of lung, pancreas, and head and neck cancer. When administered in combination with cisplatin and gemcitabine, erlotinib more effectively induced xenograft growth arrest, and this combination was more effective than either the chemotherapeutic agents or erlotinib alone (Higgins *et al*, 2004). Similar observations were previously reported regarding experimental chemosensitisation by EGFR blockade using mAbs, particularly C225. Thus, C225 (or M225) has been shown to enhance the antitumour activity of several anticancer agents in both cell cultures and human tumour xenograft models (Magné *et al*, 2002; Prewett *et al*, 2002; Balin-Gauthier *et al*, 2005). Of note, the results reported by Prewett *et al* (2002) demonstrated not only enhanced antitumour activity of C225 combined with irinotecan (CPT-11), but also that this combination was highly effective against established, CPT-11-refractory colorectal tumours. A majority of combinations between anti-EGFR drugs and cytotoxic agents result in additive and supra-additive cytotoxic effects. However, it cannot be ruled out that antagonisms may also occur with drugs not covered by these experiments.

### To the bedside

In a number of cases, preclinical studies on EGFR targeting combined with cytotoxic drugs have been confirmed clinically, the most convincing instance being the therapeutic success achieved by the cetuximab–irinotecan association in irinotecan-refractory advanced colorectal cancer patients (Cunningham *et al*, 2004). In head and neck cancer, encouraging results have been obtained recently with the association between cisplatin and cetuximab in cisplatin-refractory patients (Baselga *et al*, 2005) and also, importantly, with a combination of cetuximab and irradiation (Bonner *et al*, 2006). Although the EGFR target is present in the most frequent cancers, such as breast and prostate tumours, the benefits of anti-EGFR therapy plus cytotoxic agents are not yet apparent in these cases. More precisely, few clinical data confirm a superior clinical activity when associating anti-EGFR TKIs with cytotoxic agents. This contrasts with convincing clinical data on the benefit of combining mAbs like cetuximab with cytotoxic agents. A ray of hope comes from the erlotinib–gemcitabine combination in advanced pancreatic cancer but the benefit in terms of increased survival remains very modest in comparison with gemcitabine alone (Moore, 2005). Particularly demonstrative was the failure of the TKI–chemotherapy combinations in pretreated lung cancer patients, which was true for both gefitinib and erlotinib (Gatzemeier *et al*, 2004; Herbst *et al*, 2005). In this context, how can the discrepancies between the promising bench data and the disappointing bedside observations be explained? Apart from the classical arguments based on the obvious difficulties involved in extrapolating from experimental studies on tumour cell lines to the clinical reality; here, we will be dealing with the adequacy of the applied models and the treatment of data from preclinical experiments. We will also examine the importance of the sequence of drug combinations involving anti-EGFR drugs and cytotoxic agents.

### Back to the bench: models and limitations

To evaluate quantitatively the results of drug combinations, Chou and Talalay (1984) have proposed a method of calculation based on kinetic principles. The approach is simple and applicable whatever the dose–effect relationships (hyperbolic or sigmoidal), whatever the effects of the drugs (agonists or antagonists, mutually exclusive or not) and whatever the number of drugs involved in the combination (two or three). Many of the preclinical *in vitro* studies analysing the effects of combining EGFR-targeting drugs and chemotherapeutic compounds have been performed using the Chou and Talalay method. However, application of this method to cytostatic drugs such as those targeting EGFR may limit the significance of their final conclusion. This is mainly because, unlike true cytotoxic dose–response curves, cell proliferation inhibition leads to incomplete dose–effect curves (that is, total growth inhibition cannot be achieved) with IC_50_ values (defined as the drug concentration at the inflexion point) in the *μ*M range. These concentrations are well above the pharmacologically relevant concentrations, reflecting a partial dependency of tumour cell lines to EGFR signalling. Only very rare EGF-addicted cell lines, such as A431, with a particularly high EGFR expression can give a complete dose–effect curve with an anti-EGFR drug. Of note, the Chou and Talalay model is also not applicable for the examination of tumour growth *in vivo*, in immunodeficient mice for instance. Thus, a number of *in vivo* studies testing combinations between anti-EGFR drugs and chemotherapeutic agents have concluded that synergistic interactions have occurred without the application of a specific statistical tool to calculate the final combined effects. In experiments combining cetuximab and irinotecan, Prewett *et al* (2002) have proposed the notion of a combination ratio (CR) between expected and observed FTV, FTV being the fractional tumour volume calculated as the ratio between the mean tumour volumes of treated and untreated tumours. This simple approach has the advantage of distinguishing supra- from infra-additivity but was not used by Prewett as a strict statistical evaluation. In this respect, comparisons of Kaplan–Meier curves as used for survival analyses in patients should be encouraged; these curves could compare the times necessary for individual tumours to reach predefined volumes, and this approach would allow statistical comparisons between groups but not a strict evaluation of synergistic interactions.

Another limitation of *in vivo* combination studies is the fact that conclusions are often drawn from one or two xenograft models with a fixed schedule. A convincing illustration is provided by the paclitaxel–gefitinib combination re-examined by Solit *et al* (2005) regarding the importance of the drug association schedule. These authors made the logical hypothesis that gefitinib leading to G_1_ growth arrest of EGFR-dependent tumour cells may, following continuous administration, attenuate the effects of tubulin inhibitors – such as paclitaxel – which act primarily during mitosis. From gefitinib–paclitaxel combination studies on tumour xenograft models, they found that, in contrast to continuous administration of gefitinib, 2 days of gefitinib before paclitaxel was a most effective treatment giving significantly greater tumour regression and more frequent complete responses than the other schedule. Onn *et al* (2004) who used an orthotopic model of lung cancer provided further confirmation of an unfavourable schedule of association between continuous exposure to an EGFR TKI and paclitaxel. Three *in vivo* experiments showed a schedule-dependent efficacy of gefitinib with a better efficacy when gefitinib was administered after the cytotoxic agent, that is, in combination with cisplatin on a murine hepatocellular carcinoma (Zhu *et al*, 2005), or with gemcitabine on a head and neck carcinoma xenograft (Chun *et al*, 2006) or with docetaxel on a bladder carcinoma xenograft (Kassouf *et al*, 2006). Sequential administration of gefitinib given after chemotherapy seems to be preferable than concomitant treatment. It is thus possible that part of the failure of the gefitinib–chemotherapy combination in patients with nonsmall cell lung cancer (NSCLC) may be accounted for by an inadequate schedule of association between the anti-EGFR drug and cytotoxic agents. Reports by others (Xu *et al*, 2003a; Morelli *et al*, 2005) and ourselves (Magné *et al*, 2002) have clearly underlined, from *in vitro* studies, the importance of the sequence of association for the final antiproliferative effect of cytotoxic drugs combined with EGFR inhibitors. Thus, synergy is not always the rule and some antagonistic effects may result from inappropriate sequences between anti-EGFR drugs and cytotoxic agents as recently stressed by Davies *et al* (2006).

## A ray of hope

Recent experimental data indicate that gefitinib can restore sensitivity to topotecan in multidrug-resistant NSCLC cells overexpressing the breast cancer resistance protein (BCRP) (Nagashima *et al*, 2006). This observation is consistent with a previous report pointing out that certain TKIs, including gefitinib, interact with ABC multidrug transporter proteins with high affinity (Ozvegy-Laczka *et al*, 2004). In cells expressing ABCG2, gefitinib greatly increased the cytotoxicity of mitoxantrone. Recently, Yang *et al* (2005) showed that sensitivity to chemotherapeutic agents (paclitaxel, topotecan and doxorubicin) of multidrug resistant (MDR) cancer cells was increased in the presence of clinically relevant concentrations of gefitinib. Drug pharmacokinetics that is affected by BCRP or P-glycoprotein may be altered in the presence of gefitinib. In nontumour-bearing mice, gefitinib treatment dramatically increased the bioavailability of irinotecan after simultaneous oral administration (Stewart *et al*, 2004). Van Schaeybroeck *et al* (2005) showed that phospho-EGFR levels determine the sensitivity of human colorectal cancer cells to gefitinib alone and that chemotherapy-mediated changes in phospho-EGFR levels determine the nature of interaction between gefitinib and chemotherapy. A significant antagonism was observed between gefitinib- and oxaliplatin-induced cell death in cell lines with low basal EGFR phosphorylation levels, whereas in cell lines with high basal EGFR phosphorylation, the interaction between gefitinib and oxaliplatin was additive and often supra-additive/synergistic (Van Schaeybroeck *et al*, 2005). Similar results were obtained in human NSCLC cells (Van Schaeybroeck *et al*, 2006). However, different data were obtained for cetuximab preclinical antitumour activity (monotherapy and combination based), which was not predicted by relative total or activated EGFR tumour expression levels (Wild *et al*, 2006). A major advance for a patient selection with the objective to draw greater benefits from a combination between anti-EGFR drugs and conventional chemotherapeutic agents comes from the tumour determination of EGFR and K-Ras mutational status. In lung cancer patients, molecular studies have revealed that EGFR-activating mutations are frequently found in patients who have the best outcomes with EGFR TKIs combined with conventional chemotherapy (Bonomi *et al*, 2007). Several groups from Europe and the United States have reported that the absence of K-Ras mutation was a condition necessary to obtain a clinical response to the association between cetuximab and irinotecan in irinotecan refractory patients (Lièvre *et al*, 2008).

Another interesting perspective is the combination of anti-EGFR drugs with other targeted agents and cytotoxic treatments. We, thus, recently investigated the effects of a combination of AZD2171 – a highly potent, orally active, VEGF-signalling inhibitor – gefitinib and irradiation (Bozec *et al*, 2007). The antitumour efficacy of these treatments, administered alone or in combination, was assessed in a human head and neck tumour cell line, CAL33, established as xenografts in nude mice. Greater inhibition of tumour growth was seen with the triple combination that almost completely abolished tumour cell proliferation. Of interest, the marked irradiation-induced enhancement of the DNA-repair enzyme ERCC1 expression was totally abolished by the triple combination.

## Conclusion

Epidermal growth factor receptor-targeting agents, with their encouraging efficacy, mild toxicity profile and quality of life benefits, offer hope for patients with advanced cancer. Despite the failure of combining TKIs with chemotherapy in several large phase III clinical trials (especially in stage III NSCLC), the chemotherapy and targeted therapy combination approach is still a viable clinical research paradigm (Baselga, 2004). Future research efforts will have to be directed at identifying more efficient and effective ways of differentiating the EGFR-targeting drugs from each other, integrating these agents with conventional treatments, and finding better ways of predicting whether prolongation of life for an individual patient will be achieved.

Preclinical sequencing data are encouraging and will hopefully translate to the clinical setting. Appropriately designed clinical trials are required to define the optimum dose, schedule and sequence for these agents in combination with conventional therapies. Several clinical studies are ongoing or planned to examine sequential dosing regimens of erlotinib or gefitinib with chemotherapy in patients with various solid tumour types. The sequential approach will be tested in a US intergroup phase III study in which patients will be randomly assigned to gefitinib or placebo following chemoradiotherapy and consolidation docetaxel in patients with inoperable stage III NSCLC.

Further translational studies including examination of molecular biomarkers such as gene-expression profiling, will help us to identify critical markers and allow us to select the patients most likely to benefit from therapy with TKIs. The complexity of the ErbB signalling network and the significance of various activated downstream markers remain under intense investigation with regard to potential prognostic and predictive value.

The next clinical studies with EGFR-targeting agents should be designed with a strong translational research component to address several key questions, such as which patients are most likely to have a therapeutic benefit, what are the potential predictive factors of response or resistance to these agents and what are the best strategies for their combination with conventional and/or other targeted anticancer treatments.
